# Cholinergic innervation topography in GBA-associated *de novo* Parkinson’s disease patients

**DOI:** 10.1093/brain/awad323

**Published:** 2023-09-25

**Authors:** Sofie Slingerland, Sygrid van der Zee, Giulia Carli, Anne C Slomp, Jeffrey M Boertien, Emile d’Angremont, Nicolaas I Bohnen, Roger L Albin, Teus van Laar

**Affiliations:** Department of Neurology, University of Groningen, University Medical Center Groningen, 9713 GZ Groningen, The Netherlands; Department of Neurology, University of Groningen, University Medical Center Groningen, 9713 GZ Groningen, The Netherlands; Department of Neurology, Division of Clinical Neuropsychology, University of Groningen, University Medical Center, 9713 GZ Groningen, The Netherlands; Department of Nuclear Medicine and Molecular Imaging, University of Groningen, University Medical Center Groningen, 9713 GZ Groningen, The Netherlands; Department of Neurology, University of Groningen, University Medical Center Groningen, 9713 GZ Groningen, The Netherlands; Department of Neurology, Division of Clinical Neuropsychology, University of Groningen, University Medical Center, 9713 GZ Groningen, The Netherlands; Department of Neurology, University of Groningen, University Medical Center Groningen, 9713 GZ Groningen, The Netherlands; Department of Biomedical Sciences of Cells and Systems, University of Groningen, University Medical Center Groningen, 9713 GZ Groningen, The Netherlands; Department of Neurology, University of Michigan, Ann Arbor, MI 48109, USA; Department of Radiology, University of Michigan, Ann Arbor, MI 48109, USA; Neurology Service and GRECC, VA Ann Arbor Healthcare System, Ann Arbor, MI 48105, USA; Morris K. Udall Center of Excellence for Parkinson’s Disease Research, University of Michigan, Ann Arbor, MI 48109, USA; Parkinson’s Foundation Research Center of Excellence, University of Michigan, Ann Arbor, MI 48109, USA; Department of Neurology, University of Michigan, Ann Arbor, MI 48109, USA; Neurology Service and GRECC, VA Ann Arbor Healthcare System, Ann Arbor, MI 48105, USA; Morris K. Udall Center of Excellence for Parkinson’s Disease Research, University of Michigan, Ann Arbor, MI 48109, USA; Parkinson’s Foundation Research Center of Excellence, University of Michigan, Ann Arbor, MI 48109, USA; Department of Neurology, University of Groningen, University Medical Center Groningen, 9713 GZ Groningen, The Netherlands

**Keywords:** *GBA*, acetylcholine, Parkinson’s disease, PET, cognition

## Abstract

The most common genetic risk factors for Parkinson’s disease are *GBA1* mutations, encoding the lysosomal enzyme glucocerebrosidase. Patients with *GBA1* mutations (GBA-PD) exhibit earlier age of onset and faster disease progression with more severe cognitive impairments, postural instability and gait problems. These GBA-PD features suggest more severe cholinergic system pathologies. PET imaging with the vesicular acetylcholine transporter ligand ^18^F-F-fluoroethoxybenzovesamicol (^18^F-FEOBV PET) provides the opportunity to investigate cholinergic changes and their relationship to clinical features in GBA-PD.

The study investigated 123 newly diagnosed, treatment-naïve Parkinson’s disease subjects—with confirmed presynaptic dopaminergic deficits on PET imaging. Whole-gene *GBA1* sequencing of saliva samples was performed to evaluate *GBA1* variants. Patients underwent extensive neuropsychological assessment of all cognitive domains, motor evaluation with the Unified Parkinson’s Disease Rating Scale, brain MRI, dopaminergic PET to measure striatal-to-occipital ratios of the putamen and ^18^F-FEOBV PET. We investigated differences in regional cholinergic innervation between GBA-PD carriers and non-*GBA1* mutation carriers (non-GBA-PD), using voxel-wise and volume of interest-based approaches. The degree of overlap between t-maps from two-sample *t*-test models was quantified using the Dice similarity coefficient.

Seventeen (13.8%) subjects had a *GBA1* mutation. No significant differences were found in clinical features and dopaminergic ratios between GBA-PD and non-GBA-PD at diagnosis. Lower ^18^F-FEOBV binding was found in both the GBA-PD and non-GBA-PD groups compared to controls. Dice (*P* < 0.05, cluster size 100) showed good overlap (0.7326) between the GBA-PD and non-GBA-PD maps. GBA-PD patients showed more widespread reduction in ^18^F-FEOBV binding than non-GBA-PD when compared to controls in occipital, parietal, temporal and frontal cortices (*P* < 0.05, FDR-corrected). In volume of interest analyses (Bonferroni corrected), the left parahippocampal gyrus was more affected in GBA-PD.

*De novo* GBA-PD show a distinct topography of regional cholinergic terminal ligand binding. Although the Parkinson’s disease groups were not distinguishable clinically, in comparison to healthy controls, GBA-PD showed more extensive cholinergic denervation compared to non-GBA-PD. A larger group is needed to validate these findings. Our results suggest that *de novo* GBA-PD and non-GBA-PD show differential patterns of cholinergic system changes before clinical phenotypic differences between carriers versus non-carrier groups are observable.

## Teaser

Slingerland *et al.* examine the clinical profile and cholinergic topography of 123 newly diagnosed patients with Parkinson’s disease with and without *GBA1* mutations. PET scans reveal more extensive cholinergic denervation in *GBA1* carriers than non-carriers relative to healthy controls.

## Introduction

The most common genetic risk factors for Parkinson’s disease are heterozygous mutations of *GBA1*, encoding the lysosomal enzyme glucocerebrosidase.^1,2^ Parkinson’s disease patients with *GBA1* mutations (GBA-PD) exhibit more aggressive disease than Parkinson’s disease patients without *GBA1* mutation (non-GBA-PD).^3,4^ GBA-PD is associated with earlier and faster cognitive decline.^5^ In GBA-PD, risk of dementia is increased up to 6-fold and begins ∼5 years earlier when compared to unselected Parkinson’s disease populations.^6-9^ Neuropsychiatric symptoms are more frequently observed in GBA-PD^10,11^ and GBA-PD is associated with the postural instability and gait difficulties (PIGD) motor subtype.^5,12^ Heterogeneity of the GBA-PD phenotype is related to the type of mutation, including mild and more severe variants.^10^

Postural instability and gait problems are associated with brain impaired cholinergic systems, independent of dopaminergic deficits.^13^ Cognitive decline and cholinergic dysfunctions are interrelated.^14,15^ Previous *in vivo* Parkinson’s disease neuroimaging assessments, using acetylcholinesterase PET, demonstrated regional cholinergic synapses deficits primarily in posterior cortices.^16^ Cholinergic denervation can already be present in early Parkinson’s disease and is more widespread and severe in Parkinson’s disease dementia and dementia with Lewy bodies.^17-19^ We demonstrated previously that recently diagnosed, treatment-naïve Parkinson’s disease patients, relative to controls, show bidirectional changes in a regional expression of the vesicular acetylcholine transporter, a specific marker of presynaptic cholinergic terminals. Increased cholinergic terminal ligand binding in early Parkinson’s disease, primarily in cognitively intact subjects, suggests compensatory cholinergic upregulation in this group.^17^

GBA-PD patients often show faster progression of both motor and non-motor features with greater risks of cognitive impairments and postural instability-gait disorders, both features associated with cholinergic deficits.^4^ Analysis of cholinergic system changes in GBA-PD is needed to understand the pathophysiology of these morbid Parkinson’s disease features. There are little data on brain cholinergic systems in GBA-PD. In-depth characterization of GBA-PD is important to validate GBA-PD as a potentially distinct subtype for potential personalized treatment approaches. More detailed phenotyping of early GBA-PD is likely to provide useful prognostic information.

This study investigated the clinical phenotype and regional density of cholinergic terminals in newly diagnosed, treatment-naïve Parkinson’s disease patients, comparing GBA-PD with non-GBA-PD and healthy controls (HC) with ^18^F-fluoroethoxybenzovesamicol (^18^F-FEOBV) PET. ^18^F-FEOBV binds to the vesicular acetylcholine transporter and is a valid marker of cholinergic terminal integrity in both healthy and diseased brains.^20-22^

## Materials and methods

### Participants

One hundred and twenty-three newly diagnosed, treatment-naïve Parkinson’s disease patients and 16 healthy controls were included in the Dutch Parkinson Cohort (DUPARC) study.^23^ Inclusion criteria for patients consisted of clinically probable Parkinson’s disease diagnosis by a movement disorders specialist according to Movement Disorders Society (MDS) Clinical Diagnosis Criteria for Parkinson’s disease^24^ and with confirmed dopaminergic striatal deficits on ^18^F-3,4-dihydroxy-6-18F-fluoro-I-phenylalanine (^18^F-FDOPA) PET. The ^18^F-FDOPA scans were defined pathological by calculation of striatal-to-occipital ratios of the putamen and caudate nucleus, and compared to a normative dataset as expressed by *z*-scores. Normative data were obtained from a previous performed study,^25^ corrected for the camera used. A pathological ^18^F-FDOPA scan was defined, when the lowest (left or right) putaminal ratio was > 2 standard deviations (SD) below healthy control average (cut-off ratio: 2.18). All ^18^F-FDOPA PET scans were assessed by a nuclear medicine specialist, according to standard clinical practice of the Movement Disorders outpatient clinic of the University Medical Center Groningen. Healthy controls had a normal neurological examination and no history of neurological or psychiatric disorders. Exclusion criteria included inability to provide written informed consent, use of dopaminergic and/or (anti-)cholinergic medication and estimated low premorbid intelligence level (estimated IQ <70 on the Dutch Adult Reading test).^26^ The study was conducted according to the Good Clinical Practice guidelines and the Declaration of Helsinki. All subjects gave written informed consent and the local ethics committee approved the study.

### Genotyping

Saliva was obtained from Parkinson’s disease patients using Oragene DNA OG-500 tubes (DNA Genotek). DNA isolation, next generation sequencing (NGS) and data analysis were performed by GenomeScan B.V., Leiden, The Netherlands. Primers were selected to unambiguously sequence the functional *GBA1* gene and not the pseudogene, using long-range PCR. Fragments were amplified using PCR and sequenced using Illumina cBot and HiSeq 400, as described previously.^25^ The allelic nomenclature of the *GBA1* variants is given.

### Matching procedure

Genotyping led to unbalanced groups in terms of sample size (Table 1 and ‘Results’ section), where non-GBA-PD patients represented the majority of participants, consistent with the known epidemiology of GBA-PD mutations.^27^ Since equal-sized groups maximize statistical power,^28^ we created non-GBA-PD subgroups with identical subject numbers as the GBA-PD group. Non-GBA-PD patients were matched 1:1 for age and gender with GBA-PD subjects by case-control matching (SPSS version 28). Match tolerances were zero for gender and 2 years for age (non-GBA-PD Group 1). A second matched non-GBA-PD group was created to validate the results using a similar procedure after excluding the first already matched non-GBA-PD subjects. The match tolerances were zero for gender and 6 years for age in the second non-GBA-PD group (non-GBA-PD Group 2).

**Table 1:** Demographics and clinical characteristics of the study participants

	HC (*n* = 16)	GBA-PD (*n* = 17)	non-GBA-PD *(n* = 106)	*P*-value
Age, years^a^	65.13 (7.7)	65.2 (10.3)	64.6 (9.1)	0.961
Gender, male, *n* (% male)	11 (68.8%)	10 (58.8%)	78 (73.6%)	0.453
Educational level^b^	5.0 [5.0–6.00]	5.0 [4.0–6.0]	5.0 [4.0–6.0]	0.299
Putaminal dopaminergic striatal-to-occipital ratio, most affected side		1.79 [1.66–1.95]	1.83 [1.76–1.96]^c^	0.203
**Motor symptoms**				
Motor symptom duration, months		24.00 [8.25–30.00]^d^	24.00 [12.00–24.00]^e^	0.977
UPDRS-III, total score		29.24 (13.42)	30.54 (10.52)^f^	0.688
Motor phenotype, *n* TD/PIGD/indeterminate		6/7/4 (35%/41%/24%)	43/49/14 (41%/46%/13%)	0.535
Hoehn and Yahr stage		2.0 [1.0–2.0]	2.0 [1.0–2.0]	0.579
**Non-motor symptoms**				
MoCA, total score	28.00 [26.25–29]	27.00 [25–27]	26.00 [23–27]	**0**.**013^g^**
PD-MCI		5 (29.4%)	41 (38.7%)	0.461
*Z*-score memory		−0.06 (0.73)	−0.33 (0.61)	0.161
*Z*-score attention		−0.72 (0.66)	−0.76 (0.79)	0.853
*Z*-score executive function		−0.38 (0.72)	−0.44 (0.84)	0.774
*Z*-score language		−0.07 (0.69)	−0.03 (0.82)	0.778
*Z*-score visuospatial function		−0.11 (0.97)	−0.15 (0.99)	0.896
NMS-Quest, total score		4.0 [3.00–7.75]^h^	6.0 [3.0–9.0]^i^	0.419
HADS anxiety, total score		4.0 [1.0–6.5]	4.0 [2.0–7.0]	0.546
HADS depression, total score		2.0 [2.0–4.0]	3.0 [1.0–6.0]	0.513
RBD Quest, total score		3.00 [1.0–4.5]	3.0 [2.0–6.0]^j^	0.875
Sniffin’ sticks, total score		5.06 (2.33)	5.64 (2.56)	0.356

GBA-PD = Parkinson’s disease patients who carry *GBA1* mutations; non-GBA-PD = Parkinson’s disease patients without *GBA1* mutations; HADS = Hospital Anxiety and Depression Scale; HC = healthy controls; MoCA = Montreal Cognitive Assessment; NMS-Quest = Non-Motor Symptoms Questionnaire; PD-MCI = Parkinson’s Disease Mild Cognitive Impairment; PIGD = postural instability and gait difficulty; RBD Quest = NMS-REM Sleep Behavior Disorder Screening Questionnaire; Sniffin’ sticks = Burghart Sniffin’ Sticks 12 Tests; TD = tremor dominant; UPDRS-III = Unified Parkinson’s Disease Rating Scale part III.

^a^Mean (standard deviation).

^b^Median [Q1–Q3].

^c^Missing *n* = 2.

^d^Missing *n* = 1.

^e^Missing *n* = 8.

^f^Missing *n* = 2.

^g^ *Post hoc*: HC > GBA-PD = HC > non-GBA-PD.

^h^Missing *n* = 1.

^i^Missing *n* = 3.

^j^Missing *n* = 1.

### Clinical examination

All Parkinson’s disease participants underwent comprehensive neuropsychological assessment covering the main cognitive domains: memory, attention, executive functions, language and visuospatial abilities. A selection of outcome measures of tests and subtests of the cognitive test battery was made *a priori*, allowing implementation of level II criteria for Parkinson’s disease with mild cognitive impairment (PD-MCI; Table 2). Below-threshold performance on at least two neuropsychological tests was required for PD-MCI classification.^29,30^ Scores >1.5 SD below normative values were considered abnormal. Patients were categorized as either Parkinson’s disease with normal cognition or PD-MCI. All (sub)test scores were transformed into standardized *z*-scores based on established test-specific normative data. Test scores for the Boston naming test and the Location learning test were transformed into standardized *z*-scores based on a sample of 108 controls (58 males, ages ranging between 41 and 84 years; mean age = 64.49 years, SD = 8.08 years) collected as part of the DUPARC study. All *z*-scores within one cognitive domain were averaged to define a domain-specific *z*-score for each of the five domains. Control subjects undergoing ^18^F-FEOBV PET imaging underwent cognitive testing using the Montreal Cognitive Assessment test. Visual difficulties, colour blindness, speech problems and significant mood disorders that possibly influenced performance on the neuropsychological assessment and MCI grouping were taken into account prior to data analysis, and subjects with confounding conditions excluded.

**Table 2:** Cognitive scales applied per domain

Domain	Cognitive scale
Memory	RAVLT-Immediate recall
	RAVLT-Delayed recall
	Location learning test
Attention	Trial-Making Test-A
	Stroop-I
Visuospatial	Judgement of line orientation
Executive	Trial-Making Test-B
	Stroop-III
	Letter-fluency
Language	Boston naming test
	Semantic fluency: occupation
	Semantic fluency: animals

RAVLT = Rey Auditory Verbal Learning Test.

Clinical motor performance was examined using the MDS-revised Unified Parkinson’s Disease Rating Scale part III (UDPRS-III). Specific items from the UPDRS parts II and III were used for classification of motor phenotype, using standard criteria.^31^ Participants were classified as tremor dominant, postural instability and gait difficulty, and indeterminate motor phenotypes. Additional clinical assessments included the Hospital Anxiety and Depression Scale, Non-Motor Symptoms Questionnaire, REM Sleep Behavior Disorder Screening Questionnaire, and Burghart Sniffin’ Sticks 12 Tests.^23^ All assessors were blinded to the genetic status of the patients, as data collection was performed prior to the genetic screening.

### Image acquisition

All subjects underwent brain MRI, ^18^F-FDOPA PET and vesicular acetylcholine transporter (VAChT) PET imaging with ^18^F-FEOBV. MRI of Parkinson’s disease subjects was acquired using Siemens Magnetom Prisma 3 T MRI scanners, equipped with SENSE-8 channel head coil. For each subject, anatomical T_1_-weighted images were obtained using a sagittal 3D gradient-echo T_1_-weighted sequence with 0.9 × 0.9 × 0.9 mm acquisition. Control subjects underwent a T_1_-weighted MRI scan (3 T Intera) with 1.0 × 1.0 × 1.0 mm acquisition. ^18^F-FEOBV imaging was performed on the same day as MRI. For ^18^F-FEOBV PET imaging, participants first underwent lose-dose CT for attenuation and scatter correction using either a Biograph 40-mCT or 64-mCT (Siemens Healthcare). Both scanners were EARL certified, had the same software version and used identical acquisition and reconstruction protocols and PET detectors. Thirty-minute scans (in six 5-min frames) were acquired at 210 min post-bolus injection of ^18^F-FEOBV. ^18^F-FDOPA PET was performed after at least 6 h of fasting (4 h for diabetic patients). Participants were premedicated with carbidopa 60 min before receiving 200MBq of the FDOPA tracer, 90 min after which the PET-scan was formed on a Siemens HR+ camera.

### Image preprocessing and analysis

We visually checked all images and set the origin on the anterior commissure. Statistical parametric mapping (SPM12; Wellcome Trust Centre for Neuroimaging, London, UK) software was used to realign the PET imaging frames (six 5-min frames) within subjects to reduce the effects of subject motion during the imaging session. We co-registered ^18^F-FEOBV PET images to their corresponding T_1_-weighted volumetric scan (MRI) in native space. We then computed parametric images to reflect the distribution volume ratios (DVR) of ^18^F-FEOBV in the brain using supratentorial white matter above the ventricles as the reference region. A strong morphological erosion was applied to eliminate any spill-over effect.^32^ In addition, the Muller-Gartner partial-volume correction method was used to remove partial volume effects in the parametric PET images. PMOD version 3.8 was used to quantify ^18^F-FDOPA striatal-to-occipital ratios of the putamen.^33^

#### Volume of interest analyses

Each MRI T_1_-weighted image underwent FreeSurfer-based segmentation using the recon-all pipeline with the default settings. Cortical and subcortical labels from Deskian/Killiany and Aseg atlases parcellation were used to identify subject-specific grey matter volumes of interest (VOIs). We extracted the mean DVR values from partial volume effect corrected ^18^F-FEOBV PET parametric images in native space. We use MRI-derived VOIs and partial volume effect corrected parametric PET images registered to MRI in native space, to further mitigate partial volume effects and not introduce spill-over artefacts due to image translation from native to Montreal Neurological Institute (MNI) spaces.

#### Voxel-wise analyses

Partial volume effect corrected parametric images were normalized to a study-specific template in MNI space using high-dimensional DARTEL registration. The study-specific MNI template was obtained via the DARTEL normalization procedure on MRI T_1_-weighted images. Last, we applied an 8 mm full-width at half-maximum smoothing filter to improve the signal-to-noise ratio. A final voxel size of 2.0 × 2.0 × 2.0 mm^3^ was used.

After the described preprocessing, we assessed differences in ^18^F-FEOBV binding. First, we evaluated cholinergic topography in both GBA-PD and non-GBA-PD groups, comparing them to the topography of cholinergic terminals in healthy controls. Second, we directly compared GBA-PD and non-GBA-PD. This analysis was repeated with the second non-GBA-PD group for validation.

##### GBA-PD and non-GBA-PD versus healthy controls

We wanted to investigate the differences between control and patients’ groups to identify the lower VAChT binding (DVR significantly lower than controls) and higher VAChT binding (DVR significantly higher than controls) in each Parkinson’s disease group. We implemented two-sample *t*-test models in SPM12 for the following voxel-wise comparisons: (i) GBA-PD versus controls; and (ii) non-GBA-PD Group 1 versus controls. We entered age as a covariate of no interest in all comparisons. The significant threshold was set at *P* < 0.05, false discovery rate (FDR)-corrected at the voxel level. We used a cluster size of 100 in all analyses. We then compared the topographical similarity of the voxel-wise t-maps resulting from these comparisons (non-GBA-PD Group 1 versus Controls and GBA-PD versus Controls).

We used the Dice similarity coefficient^34,35^ to quantify the degree of overlaps between t-maps resulting from each two-sample *t*-test SPM model: t-maps of lower VAChT binding—i.e. the brain clusters significantly lower than controls—and t-maps of higher VAChT binding—i.e. the clusters significantly higher than controls. This metric measures volume overlaps between two regions divided by their mean volume. It is interpreted as follows: <0.4, poor; 0.2–0.4, fair; 0.4–0.6, moderate; 0.6–0.8, good; and >0.8, excellent agreement.

##### GBA versus non-GBA-PD comparisons

We implemented two-sample *t*-test models in SPM12 for three different comparisons: (i) GBA-PD versus non-GBA-PD whole group; (ii) GBA-PD versus non-GBA-PD Group 1; and (iii) GBA-PD versus non-GBA-PD Group 2. The threshold was set at *P* < 0.05, FDR-corrected at the voxel level.

#### Voxel-based morphometry analyses

This analysis focused on examining differences in grey matter volumes on a voxel-by-voxel basis among GBA-PD, non-GBA-PD and controls. Grey matter volumes were obtained through the segmentation of T_1_-weighted volumetric scans using SPM12. Then, the grey matter volumes were spatially normalized and modulated to a study-specific template using DARTEL normalization in SPM12. The normalized grey matter volumes were smoothed with a Gaussian kernel of 8-mm full-width at half-maximum.

Smoothed and normalized grey matter volumes of patients and healthy controls (voxel size 2 × 2 × 2 mm^3^) were statistically compared through a two-sample *t*-test SPM12 model [voxel-based morphology (VBM) analyses], correcting for the total intracranial volume and age (*P* < 0.05, cluster size 100).

### Statistical analysis

We evaluated the differences in demographic, clinical and cognitive variables and cholinergic VOIs DVR values among controls, GBA-PD and non-GBA-PD groups using ANOVA for continuous variables and χ^2^ testing for dichotomous variables. We used age as a covariate in the voxel-based and VOI analyses comparing Parkinson’s disease groups and controls. We used a multivariate generalized linear model to compare mean DVR values from the extracted VOIs among clinical groups (GBA-PD, non-GBA-PD Group 1 and Controls); age was entered as covariate of no interest. The statistical threshold was set at *P* < 0.05; Bonferroni correction was used for multiple comparisons. Direct comparisons between the GBA and non-GBA-PD groups were performed using an independent sample *t*-test or a Mann-Whitney U-test, for normally and non-normally distributed data, respectively. The striatal-to-occipital ratios of the ^18^F-FDOPA uptake were quantified by dividing the mean activity of the putamen as the VOI by the mean occipital value. Statistical analyses were performed using SPSS Statistics for Windows, Version 28.0 (IBM Statistics, USA).

## Results

### *GBA1* genetic screening

In total, 17 of the 123 (13.8%) subjects in our DUPARC cohort had a *GBA1* non-synonymous variant. Seven different mutations were identified. The E326K (six subjects), T369M (five subjects) and combined E326K/D140H (two subjects) mutations were the most frequently occurring variants. All *GBA1* exonic and splice-site variants are listed in the Supplementary material.

### Demographic and clinical evaluation

The 123 *de novo* Parkinson’s disease patients (88 males) had a mean (SD) age of 64.7 (9.2) years and a mean (SD) UPDRS-III score of 30.5 (11.0). Based on MDS PD-MCI level II criteria, 46 (37.4%) Parkinson’s disease patients were classified as PD-MCI: 5 of 17 (29.4%) GBA-PD and 41 of 123 (38.7%) non-GBA-PD. Healthy control subjects had a significantly higher Montreal Cognitive Assessment score than both Parkinson’s disease groups. Comparisons between GBA-PD and non-GBA-PD groups showed no significant differences in demographic, clinical and cognitive variables (Table 1). Also, putaminal dopaminergic striatal-to-occipital ratios of the most affected side revealed no significant difference between the Parkinson’s disease groups (Table 1). Similarly, no differences emerged comparing GBA-PD and the matched non-GBA-PD groups (Supplementary Tables 2 and 3).

### Cholinergic voxel-based comparisons

#### GBA-PD and non-GBA-PD group versus controls

##### Lower ^18^F-FEOBV binding

**Figure 1:**
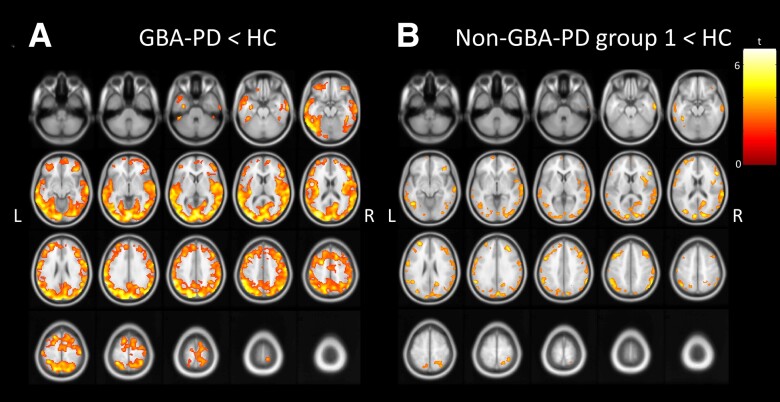
**Axial brain slices showing lower cholinergic innervation in *de novo* Parkinson’s disease patients with and without a *GBA1* mutation compared to healthy control subjects with ^18^F-FEOBV PET**. Whole brain voxel-based analyses showing significantly lower vesicular acetylcholine transporter (VAChT) binding (*P* < 0.05, FDR-corrected at voxel-level, cluster size 100) in GBA-PD, *n* = 17 (**A**) and non-GBA-PD, *n* = 17 (**B**) compared to healthy controls (HC) (*n* = 16), controlled for age. GBA-PD = Parkinson’s disease patients who carry *GBA1* mutations; non-GBA-PD = Parkinson’s disease patients without *GBA1* mutations; L = left; R = right.

Lower ^18^F-FEOBV binding was found in both GBA-PD and non-GBA-PD groups when compared to healthy controls. GBA-PD exhibited widespread lower ^18^F-FEOBV binding involving the occipital, parietal, temporal and superior and anterior frontal cortices, insula and the left parahippocampal gyrus (Fig. 1A; *P* < 0.05, FDR-corrected). Non-GBA-PD subjects exhibited lower ^18^F-FEOBV binding in bilateral middle temporal gyri, middle occipital gyri, cuneus and middle and inferior frontal gyri and right insula (Fig. 1B; *P* < 0.05, FDR-corrected). The Dice similarity coefficient (*P* < 0.05, cluster size 100) showed spatial overlap of 0.7326 (good) between the two maps (GBA-PD versus controls and non-GB-PD versus controls). This Dice coefficient indicates that the GBA-PD and non-GBA-PD group exhibit significant overlap in the distribution of diminished ^18^F-FEOBV binding, with some differences in the topography of ^18^F-FEOBV binding deficits.

##### Higher ^18^F-FEOBV binding

FDR-corrected data showed no significant voxels with higher ^18^F-FEOBV binding when GBA-PD and non-GBA-PD were compared to healthy controls. An additional exploratory FDR-uncorrected analysis was performed with a *P* < 0.05 (Supplementary Fig. 1). Higher ^18^F-FEOBV binding emerged in GBA-PD in the anterior cingulate, left lentiform nucleus and the pons compared to controls (Supplementary Fig. 1A; *P <* 0.05, uncorrected). Non-GBA-PD showed higher ^18^F-FEOBV binding than controls in both putamen and parahippocampal gyri, anterior cingulate, pons and posterior cerebellar lobe (Supplementary Fig. 1B; *P <* 0.05, uncorrected). Dice results showed a fair overlap (0.2282) when using a *P* < 0.05 and a cluster size of 100 between both binary maps.

#### GBA-PD versus non-GBA-PD groups

The results of the bidirectional whole-brain voxel-based showed no significant voxels using FDR-correction (*P* < 0.05) at the voxel level when GBA-PD was compared to non-GBA-PD group (whole, non-GBA-PD Group 1 and non-GBA-PD Group 2). Also, without FDR correction (*P* < 0.05) GBA-PD showed no higher VAChT binding when compared to non-GBA-PD groups. To gain a general view of lower cholinergic topography, the results are shown without FDR correction at the voxel level (*P* < 0.05).

##### GBA versus non-GBA whole group

**Figure 2:**
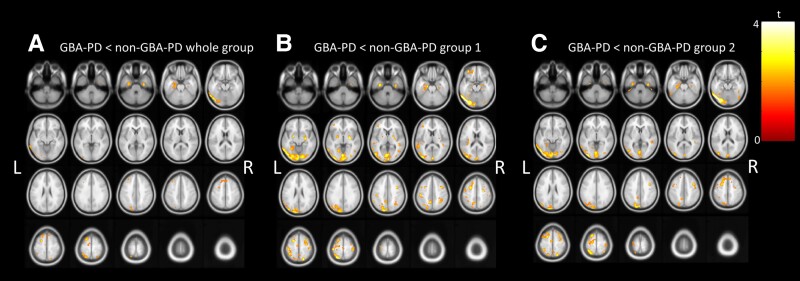
**Lower cholinergic innervation in *de novo* GBA-PD compared to (i) all non-GBA-PD within the cohort; (ii) a matched non-GBA-PD group; and (iii) a non-GBA-PD validation cohort with ^18^F-FEOBV PET**. Whole brain voxel-based analyses showing significant lower vesicular acetylcholine transporter (VAChT) binding in GBA-PD, *n* = 17 compared to non-GBA-PD whole group, *n* = 106 (**A**), compared to non-GBA-PD Group 1, *n* = 17 (**B**) and compared to non-GBA-PD Group 2, *n* = 17 (**C**), controlled for age. *P* < 0.05, uncorrected at voxel level, cluster size 100. GBA-PD = Parkinson’s disease patients who carry *GBA1* mutations; non-GBA-PD = Parkinson’s disease patients without *GBA1* mutations; L = left; R = right.

GBA-PD patients showed lower VAChT binding in the left middle and inferior occipital gyrus, both parahippocampal gyri, left inferior temporal gyrus and middle and superior frontal gyrus compared to the non-GBA-PD whole group (Fig. 2A, *P <* 0.05, uncorrected).

##### GBA versus non-GBA Group 1

Lower VAChT binding in GBA-PD compared to the matched non-GBA-PD Group 1 included lower binding in the left and right middle and inferior occipital gyrus (L > R) and left middle and superior frontal gyrus, and left and right lentiform nucleus, precuneus and parahippocampal gyrus (Fig. 2B, *P <* 0.05, uncorrected).

##### GBA versus validation group (non-GBA group 2)

In the left more than right middle and inferior occipital gyrus, the left inferior temporal gyrus, precuneus, parietal lobule, middle frontal gyrus and parahippocampus gyrus (left > right) GBA-PD showed lower VAChT binding compared to non-GBA-PD Group 2 (Fig. 2C, *P <* 0.05, uncorrected).

### Volume of interest analyses

VOI analyses showed no significant lower or higher VAChT binding regions between Parkinson’s disease groups (GBA-PD versus non-GBA-PD) after Bonferroni correction. Both GBA-PD and non-GBA-PD groups showed significant lower DVR compared to controls in the caudal middle frontal gyrus and multiple occipital parietal and temporal regions (see Table 3 for the significant VOI). GBA-PD had a significant lower ^18^F-FEOBV binding in the left parahippocampal gyrus fusiform gyrus and insula compared with controls, whereas no significant results in the non-GBA-PD to control comparison were found in these regions. Non-GBA-PD versus controls showed significantly lower VAChT binding in the left postcentral gyrus while no significant results in the comparison between GBA-PD to controls were found. Both Parkinson’s disease groups did not show any region with significantly higher VAChT binding mechanism (DVR higher than controls). All significant VOIs are shown in Table 3.

**Table 3:** Significant volume of interest of GBA-PD, non-GBA-PD and healthy control subjects after Bonferroni correction

Volume of interest	GBA-PD	Non-GBA-PD (Group 1)	HC	GBA-PD versus HC	Non-GBA-PD (Group 1) versus HC
	*n* = 17	*n* = 17	*n* = 16	*P*-value	*P*-value
Caudal middle frontal gyrus (L)	1.44 ± 0.28	1.47 ± 0.30	1.65 ± 0.18	0.017	0.042
Caudal middle frontal gyrus (R)	1.47 ± 0.25	1.49 ± 0.31	1.68 ± 0.21	0.011	0.027
Cuneus cortex (L)	1.01 ± 0.27	1.05 ± 0.28	1.31 ± 0.22	<0.001	0.002
Cuneus cortex (R)	1.00 ± 0.23	1.00 ± 0.28	1.29 ± 0.18	<0.001	<0.001
Fusiform gyrus (L)	1.32 ± 0.21	1.40 ± 0.25	1.55 ± 0.20	**0**.**001^a^**	0.060
Inferior parietal cortex (L)	1.15 ± 0.24	1.19 ± 0.19	1.47 ± 0.16	<0.001	<0.001
Inferior parietal cortex (R)	1.23 ± 0.22	1.23 ± 0.20	1.50 ± 0.12	<0.001	<0.001
Insula (L)	2.00 ± 0.26	2.02 ± 0.36	2.22 ± 0.20	**0**.**027^a^**	0.059
Insula (R)	2.03 ± 0.23	1.99 ± 0.38	2.23 ± 0.19	0.042	0.011
Lateral occipital cortex (L)	0.99 ± 0.22	1.08 ± 0.21	1.36 ± 0.19	<0.001	<0.001
Lateral occipital cortex (R)	1.05 ± 0.23	1.07 ± 0.20	1.30 ± 0.18	<0.001	<0.001
Lingual gyrus (L)	1.06 ± 0.25	1.11 ± 0.25	1.29 ± 0.15	<0.001	0.005
Lingual gyrus (R)	1.04 ± 0.23	1.06 ± 0.26	1.26 ± 0.16	0.001	0.003
Middle temporal gyrus (L)	1.44 ± 0.23	1.45 ± 0.20	1.67 ± 0.26	0.003	0.008
Middle temporal gyrus (R)	1.47 ± 0.23	1.47 ± 0.24	1.69 ± 0.24	0.018	0.017
Parahippocampal gyrus (L)	1.22 ± 0.23	1.31 ± 0.31	1.40 ± 0.24	**0**.**045^a^**	0.706
Pericalcarine cortex (L)	1.08 ± 0.36	1.11 ± 0.34	1.50 ± 0.23	<0.001	<0.001
Pericalcarine cortex (R)	1.13 ± 0.30	1.08 ± 0.27	1.46 ± 0.21	<0.001	<0.001
Postcentral gyrus (L)	1,44 ± 0.38	1.41 ± 0.40	1.66 ± 0.22	0.055	**0**.**026^b^**
Postcentral gyrus (R)	1.42 ± 0.37	1.40 ± 0.42	1.71 ± 0.26	0.013	0.007
Precuneus (L)	1.29 ± 0.35	1.32 ± 0.42	1.50 ± 0.24	0.010	0.026
Precuneus (R)	1.29 ± 0.32	1.29 ± 0.47	1.50 ± 0.29	0.006	0.007
Superior parietal cortex (L)	1.01 ± 0.24	1.03 ± 0.23	1.27 ± 0.17	<0.001	0.001
Superior parietal cortex (R)	1.04 ± 0.23	1.03 ± 0.23	1.26 ± 0.19	0.004	0.003
Superior temporal cortex (L)	1.54 ± 0.29	1.56 ± 0.29	1.82 ± 0.20	0.003	0.007
Superior temporal cortex (R)	1.60 ± 0.32	1.58 ± 0.34	1.85 ± 0.20	0.022	0.012
Supramarginal gyrus (L)	1.37 ± 0.29	1.39 ± 0.25	1.66 ± 0.15	<0.001	<0.001
Supramarginal gyrus (R)	1.46 ± 0.28	1.42 ± 0.31	1.68 ± 0.15	0.013	0.004
Transverse temporal cortex (L)	1.74 ± 0.67	1.79 ± 0.52	2.32 ± 0.36	0.002	0.006
Transverse temporal cortex (R)	1.73 ± 0.58	1.73 ± 0.61	2.28 ± 0.38	0.004	0.004

GBA-PD = Parkinson’s disease patients who carry *GBA1* mutations; non-GBA-PD = Parkinson’s disease patients without *GBA1* mutations; HC = healthy controls; L = left; R = right.

^a^GBA-PD showed significantly lower vesicular acetylcholine transporter (VAChT) binding compared to healthy controls in this VOI, whereas non-GBA-PD did not.

^b^Non-BA-PD showed significant lower VAChT binding compared to healthy controls in this VOI, whereas non-GBA-PD did not.

### Voxel-based morphometry analyses

VMB analyses revealed no significant reduced grey matter brain volume between GBA-PD and non-GBA-PD Group 1 alongside the control group (*P* < 0.05, FDR corrected, cluster size 100). Therefore, results are shown without FDR correction at the voxel level (*P* < 0.05, cluster size 100) (Supplementary Fig. 2).

## Discussion

We investigated clinical features and regional cholinergic terminal changes in *de novo* Parkinson’s disease subjects with and without relevant *GBA1* mutations. To our knowledge, this is the first assessment of regional cholinergic innervation in GBA-PD. We did not find any significant differences in demographics, clinical motor or non-motor characteristics between GBA-PD and non-GBA-PD at the time of diagnosis. Although the two Parkinson’s disease groups were not distinguishable by clinical features, *GBA1* mutation carriers showed more extensive cholinergic denervation—involving more temporo-parietal regions—than non-carriers compared to healthy controls. GBA-PD directly compared to non-GBA-PD revealed lower VAChT binding in the same regions, however these results were only statistically significant before correction for multiple comparisons. Our results suggest that *GBA1* mutations are associated with cholinergic deficits and more widespread neurodegeneration in early disease.

In our cohort, 13.8% of Parkinson’s disease patients were *GBA1* mutation carriers, consistent with recent large nationwide genetic screening of more than 3000 participants in The Netherlands.^27^ Whole-gene *GBA1* sequencing revealed that the majority (65%) of our GBA-PD patients have the E326K and T369M variants. These are the most common *GBA1* variants in The Netherlands.^27^ These variants are associated with Parkinson’s disease but not with Gaucher’s disease and normally present with a milder phenotype than other *GBA1* mutations associated with Parkinson’s disease.^36^ The prevalence of these variants in our cohort might explain why we did not find differences in the age of onset between Parkinson’s patients with and without *GBA1* mutations.^1,37^ Early age of disease onset is mainly observed in carriers of severe *GBA1* variants.^10,38^

As previously reported,^39^ GBA-PD and non-GBA-PD were clinically indistinguishable at the time of diagnosis. Previous studies also did not find significant differences in motor scores at early disease stages.^3,39,40^ Although higher prevalence of PIGD motor phenotype was documented in GBA-PD,^41^ this was not found in our GBA-PD group. These discrepancies might be due to our limited sample of *GBA1* patients, the prevalence of mild mutations and evaluation at diagnosis. Differences in motor severity between GBA and non-GBA-PD seem to emerge during later disease stages.^5^ Longitudinal assessment will provide more information on differences in disease progression between GBA-PD and non-GBA-PD carriers.

We found comparable neuropsychological performance in GBA-PD carriers and non-GBA-PD. These findings are consistent with prior studies describing GBA-PD at early disease stages.^3,39,42,43^ Previous longitudinal studies suggest diverging rates of cognitive deterioration ∼3 years after diagnosis.^39,43^

Both our voxel-wise and VOI-based analyses showed that cholinergic terminal deficits were more pronounced in GBA-PD than in non-GBA-PD. Although regional overlap exists, GBA-PD patients had more extensive deficits in parieto-temporal regions. This denervation topography is consistent with previous cholinergic imaging studies, which described predominantly posterior and temporal cortical binding differences between Parkinson’s disease patients and healthy controls.^16,21,44,45^ These studies generally focused on more advanced Parkinson’s disease subjects, though one small study using acetylcholinesterase PET identified some Parkinson’s disease subjects with early loss of cholinergic synapses.^44^ Our results suggest that GBA-PD exhibits accelerated degeneration of especially nucleus basalis of Meynert (Ch4) projections neurons.

VOI analysis results showed significantly lower ^18^F-FEOBV binding in various occipital, temporal and parietal regions in both Parkinson’s disease groups compared to controls. In addition, GBA-PD had a significant lower ^18^F-FEOBV binding than non-GBA-PD in mainly the left parahippocampal gyrus when compared with controls. Previous research found that executive function in Parkinson’s disease is associated with parahippocampal connectivity and dysfunction in paralimbic cholinergic networks was associated with Parkinson’s disease dementia.^46,47^

In the present study, we did not find significantly higher ^18^F-FEOBV binding in either the GBA-PD or non-GBA-PD group when compared to controls, as well as in the direct Parkinson’s disease group comparisons. We previously described significantly higher ^18^F-FEOBV binding in several regions in *de novo* Parkinson’s disease subjects, suggesting compensatory upregulation of cholinergic systems at early disease phases.^17^ It is possible that this discrepancy in outcome is due to the limited sample sizes in our study. Future research with a larger group of patients could better demonstrate whether GBA-PD shows a distinct altered cholinergic innervation pattern compared to non-GBA-PD.

Relevant *GBA1* mutations are related to loss of glucocerebrosidase activity and lysosomal dysfunction. Parkinson’s disease is a heterogeneous syndrome and efforts to subtype Parkinson’s disease using clinical measures are largely sterile.^48^ An alternative approach is to subtype Parkinson’s disease based on pathogenic mechanisms.^49^ GBA-PD would potentially represent one such subtype. It is possible that early basal forebrain cholinergic projection system degeneration might be characteristic of a lysosomal Parkinson’s disease subtype. Application of ^18^F-FEOBV imaging to subjects with other potential forms of lysosomal Parkinson’s disease and discovery of early, widespread basal forebrain cholinergic projection system degeneration would support the existence of a lysosomal Parkinson’s disease subtype.^50^ A recent study found cholinergic basal forebrain terminal loss in newly diagnosed dementia with Lewy bodies using ^18^F-FEOBV PET.^18^*GBA1* mutations are also associated with dementia with Lewy bodies and carriers often present with a more severe phenotype.^51^ Although no cognitive or other clinical differences were observed between the GBA-PD and non-GBA-PD groups, the distinct cholinergic topography, characterized by more widespread cholinergic denervation and limited upregulation, may be a predictor of a more aggressive clinical trajectory in GBA-PD. Longitudinal assessment is needed to explore this possibility.

As far as we know, this is the first study that assessed cholinergic system changes using PET imaging in GBA-PD *in vivo*. Previous studies using different imaging techniques reported reduced cortical activity and reduced cerebral blood flow in parietal and occipital cortices.^9,52^ Recent research also suggested cholinergic loss in *de novo* GBA-PD; however, this was based on VBM analyses of MRI T_1_-weighted images and not with a specific cholinergic tracer.^53^ A comprehensive review on neuroimaging in GBA-PD assessed all imaging techniques used to date in cross-sectional or longitudinal studies to differentiate GBA-PD from non-GBA-PD.^54^ In line with our results, previous nigrostriatal imaging studies reported mostly no differences^52,55,56^; however, contrasting findings were also found varying from reduced^9,36^ to increased dopaminergic levels.^57^ Differences in sample size, stage of Parkinson’s disease and methods used for genotyping in these studies were suggested as a possible explanation for contrasting results.^54^ A fluorodeoxyglucose (FDG)-PET study showed a more typical expression of cortical glucose metabolism—Parkinson’s disease-related pattern (PDRP)—in GBA-PD compared to non-GBA-PD, even in the absence of statistically significant clinical differences.^36^ The PDRP includes hypometabolism in the parieto-occipital cortex and therefore, we found, resembles the topography of cholinergic deficits in GBA-PD.

In this study, indications of a distinct cholinergic topography were found when directly comparing GBA-PD to non-GBA-PD; however, results of the GBA-PD versus the non-GBA-PD group comparison are based on a relatively small sample. The dual-group comparison suggests a consistent pattern but it remains possible that other factors may play a role. For example, it is possible that both the GBA-PD as well as the non-GBA-PD groups are quite heterogenous, including Parkinson’s disease patients with mild and severe subtypes. It should be noted that we only performed whole-gene *GBA1* screening and did not screen for other Parkinson’s disease-related mutations in this study. Besides *GBA1*, other (genetic) factors are also known as predictors of cognitive deterioration in Parkinson’s disease, such as variants in the apolipoprotein E, microtubule-associated protein tau and α-synuclein loci.^58-60^ Some of the non-GBA-PD might be secondary to other lysosomal defects. Also, increased acetylcholinesterase activity, suggesting upregulation of cholinergic neurotransmission, has been reported in carriers of leucine repeat kinase 2 mutations with prodromal Parkinson’s disease.^15,61^ The expected heterogeneity of both Parkinson’s disease groups, besides *GBA1* status, could be an important factor confounding the direct comparison between GBA-PD and non-GBA-PD.

### Strengths and limitations

A major strength of this study is the unbiased inclusion of *de novo* Parkinson’s disease subjects. Genetic screening was performed after the collection of the clinical data and therefore, all assessors were blinded to the genetic status of the patients. Our study included an extensive and detailed subject assessment, including extensive neuropsychological test battery, clinical assessment, putaminal dopaminergic evaluation and the use of ^18^F-FEOBV PET. The extensive cognitive assessment in this study allowed for type II classification of PD-MCI^29^ and domain-specific assessment of cognitive performance. Previous *in vivo* imaging studies of brain cholinergic systems in Parkinson’s disease mostly relied on PET acetylcholinesterase substrate tracers, which are indirect markers of cholinergic terminal integrity because acetylcholinesterase may have both pre- and post-synaptic expressions and its activity may vary over the course of the disease.^62^ The big advantage of ^18^F-FEOBV PET imaging is the strict correlation with presynaptic cholinergic terminals.^21,63^ A final strength is the use of a validation cohort comparing GBA-PD with a second matched non-GBA-PD group to validate our results.

A limitation of this study is the relatively small sample size of the GBA-PD group. Due to the heterogeneity within Parkinson’s subjects in general, significant differences might have been missed, due to limited power. Future studies with larger sample sizes would allow for more detailed stratification, for example, by mutation or sex, which will improve our understanding of the cholinergic system pathologies of GBA-PD.

Furthermore, dopaminergic striatal-to-occipital ratios comparing GBA and non-GBA Parkinson’s disease were calculated using ^18^F-FDOPA PET. Previous studies suggested that ^18^F-FDOPA slightly underestimated the severity of dopaminergic degeneration due to homeostatic upregulation of the aromatic L-amino acid decarboxylase enzyme in early disease stages.^64^ Therefore, the calculated ratios might be mildly undervalued. However, we do not expect any effect on the results between GBA-PD and non-GBA-PD.

The cross-sectional design does not allow for longitudinal assessment of cholinergic innervation changes. Further research should enhance our understanding of the role of cholinergic dysfunction in GBA-PD through longitudinal follow-up. Clarification of the relationship between cholinergic deficits and characterization of GBA-PD may be useful for its classification as a possible relevant aetiological, pathophysiological and clinical Parkinson’s disease subtype.

## Conclusion

The results of this study provide the first data on the cholinergic systems in GBA-PD subjects. This study demonstrates evidence of deficits in cortical cholinergic innervation in *de novo* Parkinson’s disease, with and without *GBA1* mutations. At the time of diagnosis, despite a similar clinical profile, *GBA1* mutation carriers showed more extensive cholinergic denervation than non-GBA-PD patients. This distinct cholinergic deficit topography suggests more advanced cholinergic degeneration in GBA-PD, potentially contributing to the faster disease progression of this subset of Parkinson’s disease subjects.

## Supplementary Material

awad323_Supplementary_Data

## Data Availability

The data that support the findings of this study are available from the corresponding author, upon reasonable request.
