# Supplementary material for: Cholinergic innervation topography in GBA-associated de novo Parkinson’s disease patients
Source: Brain. 2023 Sep 25;147(3):900–10. doi: 10.1093/brain/awad323 (PMC10907081; doi:10.1093/brain/awad323)
Supplement: awad323_Supplementary_Data [file awad323_supplementary_data.pdf]

Supplementary material

Supplementary table 1: Overview of *GBA1* variants.

**DNA sequencing of complete human *GBA1*-gene, using Illumina NovaSeq6000 sequencing.**<br>The GBA1 allelic names are given, excluding the 39 amino acid signalling peptide.			
**Number of subjects**	***GBA1* mutations (allelic name)**	**Type of mutation**	**Position Chr1**
**5**	p.T369M	missense variant & splice region variant	chr1:155206037:A
**6**	p.E326K	missense variant	chr1:155206167:T
**2**	p.E326K;p.D140H	missense variant;missense variant	chr1:155206167:T;chr1:155208361:G
**1**	p.V460=;p.A456P	synonymous variant;missense variant	chr1:155204994:G;chr1:155205008:G
**1**	p.L444P;p.V460=; p.A456P	missense variant;synonymous variant;missense variant	chr1:155205043:G;chr1:155204994:G; chr1:155205008:G
**1**	p.N370S;p.R329C;p.K346E	missense variant & splice region variant;missense variant;missense variant	chr1:155205634:C;chr1:155206158:A;chr1:155206107:C
**1**	p.F213V	missense variant	chr1:155207932:C

Supplementary table 2: Demographics and Clinical Characteristics of the study participants, comparing HC, GBA-PD and non-GBA-PD.

	HC (*n* = 16)	GBA-PD (*n* = 17)	non-GBA-PD (*n* = 17)	*P*-value
**Age y[a]**	65.13 (7.70)	65.18 (10.27)	65.35 (9.94)	0.960
**Gender, male n (% male)**	11 (68.8%)	10 (58.8%)	10 (58.8%)	0.807
**Educational level[b]**	5.0 [4.0-6.0]	5.0 [4.0-6.0]	5.0 [4.0-5.0]	0.110
**Putaminal dopaminergic striatal-to-occipital ratio, most affected side**		1.79[1.67-1.95]	1.93[1.80-1.99]*	0.087
Motor symptoms				
**Motor symptom duration**		24.00 [8.25-30.00]**	12.00 [6.50-22-00]◊	0.151
**UPDRS-III, total score**		29.24 (13.42)	32.76 (9.86)	0.325
**Motor phenotype, n TD/PIGD/indeterminate**		6/7/4 (35%/41%/24%)	8/7/2 (47%/41%/12%)	0.618
**Hoehn and Yahr stage**		2.00 [1.00-2.00]	2.00 [2.00-2.00]	0.339
Non-motor symptoms				
**MoCA, total score**	27.44 (2.10)	25.76 (2.82)	25.00 (3.46)	0.054
**PD-MCI**		5 (29.4%)	5 (29.4%)	1.000
**Z-score memory**		-0.06(0.73)	-0.28 (0.75)	0.393
**Z-score attention**		-0.72 (0.66)	-0.48 (0.84)	0.362
**Z-score executive function**		-0.38 (0.72)	-0.22 (0.83)	0.552
**Z-score language**		-0.07 (0.69)	-0.26 (0.98)	0.560
**Z-score visuospatial function**		-0.11 (0.97)	-0.17 (0.97)	0.859
**NMS-Quest, total score**		4.0 [3.00-7.75]§	6.00 [4.00-8.50]	0.337
**HADS anxiety, total score**		4.06 (3.07)	4.29 (2.47)	0.335
**HADS depression, total score**		2.00 [2.00-4.00]	5.00 [1.00-7.00]	0.245
**RBD Quest, total score**		3.00 [1-4.50]	2.00 [1.00-6.00]	0.819
**Sniffin' sticks, total score**		5.06 (2.33)	5.64 (2.56)	0.356

Abbreviations: UPDRS-III, Movement Disorders Society Unified Parkinson's Disease Rating Scale part III; TD, Tremor Dominant; PIGD, Postural Instability and Gait Difficulty; MoCA, Montreal Cognitive Assessment; PD-MCI, Parkinson's Disease Mild Cognitive Impairment; NMS-Quest, Non-Motor Symptoms Questionnaire; HADS, Hospital Anxiety and Depression Scale; RBD Quest, REM Sleep Behaviour Disorder Screening Questionnaire; Sniffin' sticks, Burghart Sniffin' Sticks 12 Tests

a. mean (standard deviation); b. median [Q1- Q3]; * missing *n* = 1, ** missing *n* = 1, ◊ missing *n* = 4, § missing *n* = 1

Supplementary table 3: Demographics and Clinical Characteristics of the study participants, comparing HC, GBA-PD and second non-GBA-PD group 2.

	HC (*n* = 16)	GBA-PD (*n* = 17)	non-GBA-PD (*n* = 17)	*P*-value
**Age y[a]**	65.13(7.70)	65.18 (10.27)	64.65 (7.78)	0.986
**Gender, male n (% male)**	11 (68.8%)	10 (58.8%)	10 (58.8%)	0.817
**Educational level[b]**	5.0 [5.0-6.0]	5.0 [4.0-6.0]	5.0 [4.0-6.0]	0.445
**Putaminal dopaminergic striatal-to-occipital ratio, most affected side**		1.79[1.67-1.95]	1.83[1.73-1.98]	0.278
Motor symptoms				
**Motor symptom duration**		24.00 [8.25-30.00]*	24.00 [10.25-28.50]**	0.406
**UPDRS-III, total score**		29.24 (13.42)	30.47 (11.86)	0.778
**Motor phenotype, n TD/PIGD/indeterminate**		6/7/4 (35%/41%/24%)	6/9/2 (35%/53%/12%)	0.639
**Hoehn and Yahr stage**		2.00 [1.00-2.00]	2.00 [1.50-2.00]	0.205
Non-motor symptoms				
**MoCA, total score**	27.44 (2.10)	25.76 (2.82)	25.35 (3.08)	**0.229**
**PD-MCI**		5 (29.4%)	3 (17.65%)	0.434
**Z-score memory**		-0.06(0.73)	-0.27 (0.51)	0.348
**Z-score attention**		-0.72 (0.66)	-0.80 (0.76)	0.742
**Z-score executive function**		-0.38 (0.72)	-0.44 (0.82)	0.814
**Z-score language**		-0.07 (0.69)	0.08 (0.86)	0.527
**Z-score visuospatial function**		-0.11 (0.97)	0.04 (0.85)	0.635
**HADS anxiety, total score**		4.06 (3.07)	4.18 (2.48)	0.903
**HADS depression, total score**		2.00 [2.00-4.00]	3.00 [1.00-4.00]	0.847
**RBD Quest, total score**		3.00 [1-4.50]	2.00 [2.00-5.50]	0.861
**Sniffin' sticks, total score**		5.06 (2.33)	6.00 (2.65)	0.279

Abbreviations: UPDRS-III, Unified Parkinson's Disease Rating Scale part III; TD, Tremor Dominant; PIGD, Postural Instability and Gait Difficulty; MoCA, Montreal Cognitive Assessment; PD-MCI, Parkinson's Disease Mild Cognitive Impairment; NMS-Quest, Non-Motor Symptoms Questionnaire; HADS, Hospital Anxiety and Depression Scale; RBD Quest, REM Sleep Behaviour Disorder Screening Questionnaire; Sniffin' sticks, Burghart Sniffin' Sticks 12 Tests

a. mean (standard deviation); b. median [Q1- Q3]; * missing *n* = 1, ** missing *n* = 1

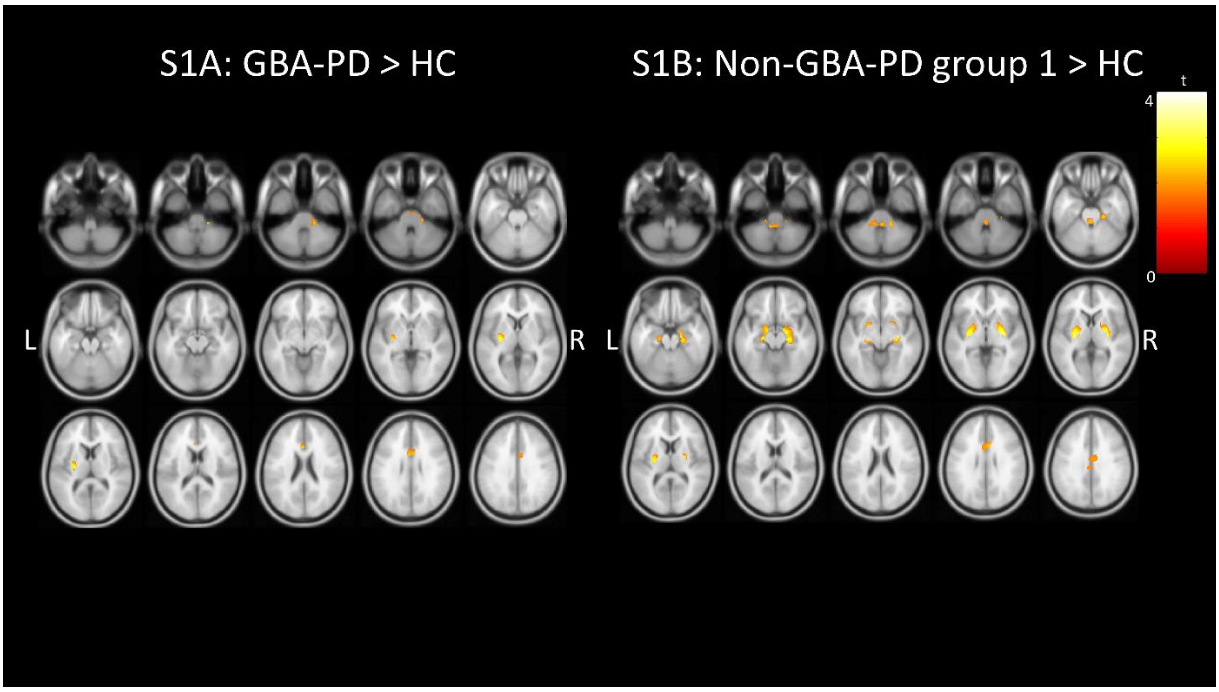

Supplementary Figure 1: Whole brain voxel-based analyses showing significant higher VAChT binding ($P < 0.05$, uncorrected at voxel level, cluster size 100) in GBA-PD, $n = 17$ (S1A) and non-GBA-PD, $n = 17$ (S1B) compared to HC ($n = 16$), controlled for age. L, left; R, right.

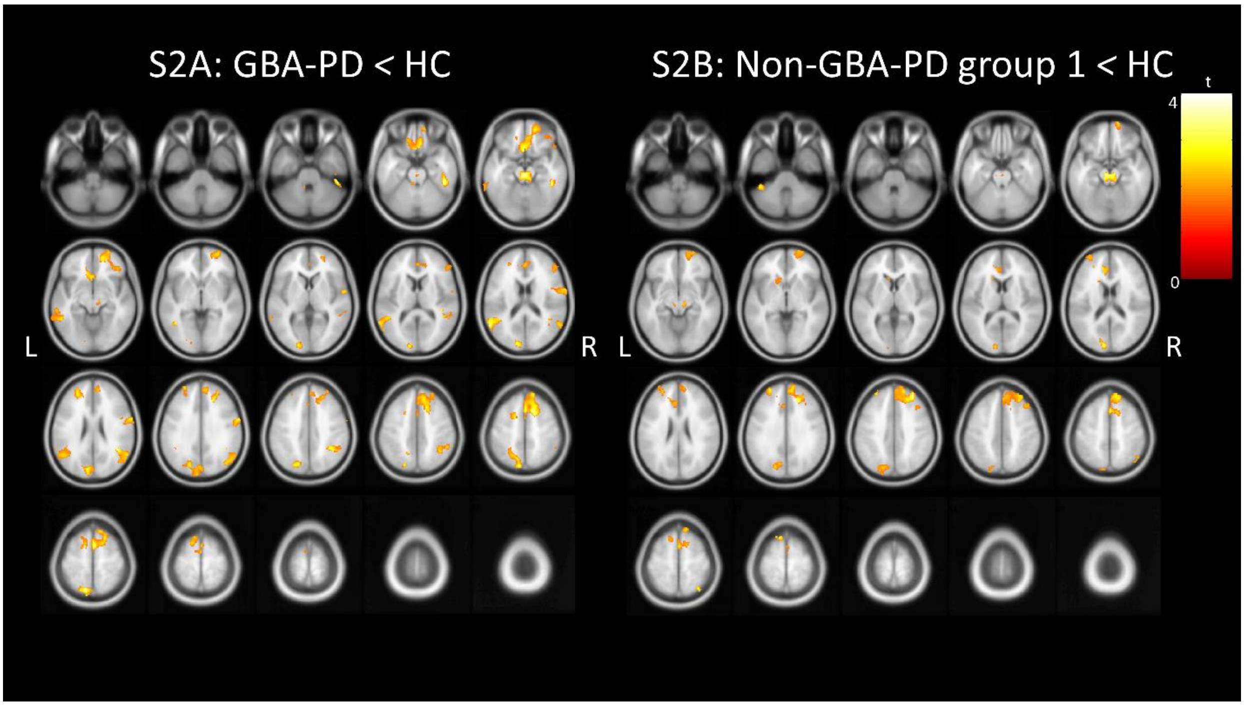

Supplementary Figure 2: Voxel-based morphometry analyses showing significant lower grey matter brain volume ($P < 0.05$, uncorrected at voxel level, cluster size 100) in GBA-PD, $n = 17$ (S2A) and non-GBA-PD, $n = 17$ (S2B) compared to HC ($n = 16$). L, left; R, right.
